# Supplementary material for: Modeling glioblastoma heterogeneity as a dynamic network of cell states
Source: Mol Syst Biol. 2021 Sep 16;17(9):e10105. doi: 10.15252/msb.202010105 (PMC8444284; doi:10.15252/msb.202010105)
Supplement: Supplementary file 5 — Source Data for Figure 3 [file MSB-17-e10105-s001.zip › Figure3A_sourcedata/GSEA_3065/nosigdb_state4.GseaPreranked.1622718430994/DOROSZKO_U3180MG_CLUSTER_5.html]

Details for gene set DOROSZKO\_U3180MG\_CLUSTER\_5[GSEA]

|  || Dataset | state4 |
| Phenotype | NoPhenotypeAvailable |
| Upregulated in class | na\_pos |
| GeneSet | DOROSZKO\_U3180MG\_CLUSTER\_5 |
| Enrichment Score (ES) | 0.5938075 |
| Normalized Enrichment Score (NES) | 1.9059354 |
| Nominal p-value | 0.0028571428 |
| FDR q-value | 0.0033394434 |
| FWER p-Value | 0.027 |
Table: GSEA Results Summary

  

Fig 1: Enrichment plot: DOROSZKO\_U3180MG\_CLUSTER\_5      
 Profile of the Running ES Score & Positions of GeneSet Members on the Rank Ordered List

  

| PROBE | GENE SYMBOL | GENE\_TITLE | RANK IN GENE LIST | RANK METRIC SCORE | RUNNING ES | CORE ENRICHMENT || 1 | GFAP |  |  | 0 | 0.995 | 0.1583 | Yes |
| 2 | ID3 |  |  | 2 | 0.730 | 0.2742 | Yes |
| 3 | CRYAB |  |  | 10 | 0.494 | 0.3516 | Yes |
| 4 | ID2 |  |  | 56 | 0.292 | 0.3909 | Yes |
| 5 | ID1 |  |  | 84 | 0.261 | 0.4281 | Yes |
| 6 | LGALS3 |  |  | 89 | 0.259 | 0.4686 | Yes |
| 7 | CYSTM1 |  |  | 108 | 0.244 | 0.5046 | Yes |
| 8 | PLA2G16 |  |  | 110 | 0.244 | 0.5432 | Yes |
| 9 | TRIM47 |  |  | 175 | 0.212 | 0.5667 | Yes |
| 10 | PLP1 |  |  | 330 | 0.169 | 0.5689 | Yes |
| 11 | MT1X |  |  | 341 | 0.167 | 0.5938 | Yes |
| 12 | GAP43 |  |  | 918 | 0.094 | 0.5164 | No |
| 13 | AIF1L |  |  | 1119 | 0.081 | 0.4971 | No |
| 14 | SLC4A4 |  |  | 1160 | 0.078 | 0.5031 | No |
| 15 | PDLIM4 |  |  | 1446 | 0.064 | 0.4677 | No |
| 16 | NRN1 |  |  | 1610 | 0.058 | 0.4508 | No |
| 17 | CD44 |  |  | 2687 | 0.031 | 0.2832 | No |
| 18 | IDI1 |  |  | 2749 | 0.030 | 0.2781 | No |
| 19 | NAMPT |  |  | 3040 | 0.024 | 0.2354 | No |
| 20 | LPL |  |  | 3121 | 0.023 | 0.2262 | No |
| 21 | S100A11 |  |  | 3436 | 0.014 | 0.1781 | No |
| 22 | GJA1 |  |  | 4123 | -0.017 | 0.0708 | No |
| 23 | ANKH |  |  | 4600 | -0.051 | 0.0026 | No |
| 24 | AEBP1 |  |  | 4733 | -0.062 | -0.0086 | No |
| 25 | ANXA1 |  |  | 5538 | -0.135 | -0.1161 | No |
| 26 | SRPX |  |  | 5662 | -0.154 | -0.1114 | No |
| 27 | LDLR |  |  | 5843 | -0.194 | -0.1095 | No |
| 28 | EMP1 |  |  | 6166 | -0.357 | -0.1044 | No |
| 29 | POSTN |  |  | 6256 | -0.753 | 0.0011 | No |
Table: GSEA details [plain text format]

  

Fig 2: DOROSZKO\_U3180MG\_CLUSTER\_5: Random ES distribution      
 Gene set null distribution of ES for **DOROSZKO\_U3180MG\_CLUSTER\_5**
